# Adult Colocolic Intussusception Secondary to a Colonic Lipoma: A Case Report

**DOI:** 10.7759/cureus.88694

**Published:** 2025-07-24

**Authors:** Ahmed R Zubi, Nabeel Atiyah

**Affiliations:** 1 General Surgery, Indira Gandhi Memorial Hospital, Malé, MDV; 2 Internal Medicine, University of Tripoli, Tripoli, LBY

**Keywords:** abdominal imaging, abdominal pain, adult intussusception, case report, colonic lipoma, segmental colectomy

## Abstract

Adult intussusception is uncommon and usually presents with nonspecific symptoms. Cross-sectional imaging is extremely important for diagnosis. In adults, a lead point is often present and indicates the underlying cause, which can be benign or malignant. Therefore, the primary treatment is surgical resection. Colonic lipoma, although rare, is a frequent benign lesion that leads to colocolic intussusception.

This report describes a 42-year-old woman who presented with 12 days of intermittent abdominal pain and no other associated symptoms. After multiple emergency department (ED) visits, her symptoms were initially attributed to constipation, as physical examination revealed only mild right lower quadrant tenderness, routine blood tests were within normal range, and abdominal X-ray (AXR) showed stool loading in the right colon. Subsequent cross-sectional imaging revealed a colocolic intussusception caused by a 5 cm submucosal lipoma acting as the lead point. She underwent open segmental colectomy with a primary side-to-side stapled anastomosis and was discharged on postoperative day eight without complications. Histopathology confirmed the presence of a lipoma and an incidental serrated adenoma.

This case highlights the diagnostic challenge and the importance of cross-sectional imaging in patients presenting with nonspecific abdominal pain. Intussusception should be considered in the differential diagnosis of adults with abdominal pain. Computed tomography (CT) is essential for timely diagnosis, and surgical resection remains the mainstay of treatment, particularly in colocolic involvement.

## Introduction

Abdominal pain is a common symptom with a broad differential diagnosis. While often benign, it requires thorough evaluation as it may signal serious pathology [1,2]. While intussusception occurs infrequently in adults, its diagnosis is often delayed due to nonspecific symptoms such as intermittent abdominal pain, nausea, vomiting, diarrhea, or hematochezia [2,3]. Some patients may present acutely with bowel obstruction [4-6]. Diagnostic imaging, particularly computed tomography (CT), plays a crucial role in evaluation, underscoring the importance of radiographic assessment in patients with vague abdominal symptoms [1,2,7]. Unlike pediatric cases, adult intussusception more frequently involves a lead point, which is neoplastic in approximately 60% of cases (either benign or malignant) [1,2,8]. Other potential causes include adhesions, intestinal ulcers, infections, Crohn's disease, Meckel's diverticulum, and idiopathic factors [1,2]. Consequently, the possibility of malignancy remains a significant concern [7].

Adult intussusception can be classified into five anatomical types: enteroenteric, ileocolic, ileocecal, colocolic, and sigmoidorectal. Among these, colocolic intussusception accounts for 11-21% of cases and carries a higher malignancy risk than small bowel involvement [1-3,7,8]. Unlike in pediatric cases, pneumatic or hydrostatic reduction is not recommended for adults [2,5]. Surgical intervention is clearly indicated for patients with signs of peritonitis, bowel obstruction, or identifiable lead points and may be performed via open or minimally invasive approaches [2,5]. For stable patients with colocolic intussusception, colonoscopy may be considered to evaluate the lead point and assess malignancy risk [1]. A conservative approach has been described for stable cases of enteroenteric intussusception without lead points, as spontaneous reduction may occur [9,10].

We present an uncommon case of colocolic intussusception secondary to a colonic lipoma in a middle-aged woman, along with a comprehensive discussion of the initial evaluation, disease characteristics, and treatment options.

## Case presentation

A 42-year-old woman presented to the emergency department (ED) with a four-day history of vague abdominal pain without vomiting or other associated symptoms. A systematic review revealed only a history of chronic constipation. Her past medical history was unremarkable, with no family history of colorectal or other malignancies. Physical examination revealed only mild right lower quadrant tenderness without guarding or rebound tenderness and no palpable mass. Her vital signs were within normal range, and she was overweight. Since her pain improved spontaneously, routine laboratory tests and an abdominal X-ray (AXR) were ordered, with follow-up scheduled for the next day.

**Figure 1:**
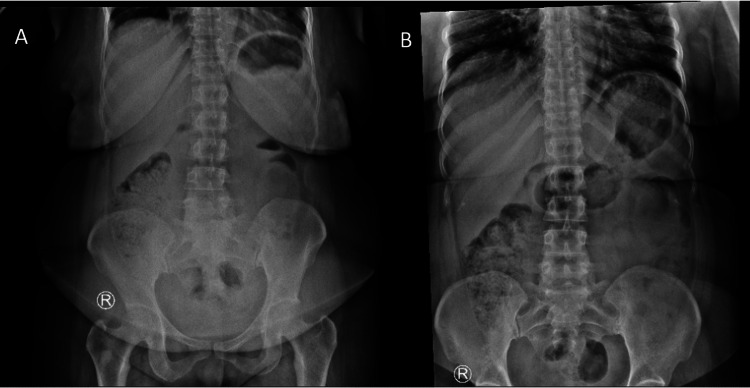
First presentation abdominal X-ray (A) Erect; (B) Supine

On follow-up evaluation (day five after initial pain onset), she reported severe, intermittent abdominal pain localized to the epigastrium and right side, although still without any other associated symptoms. Laboratory results were unremarkable except for a mildly elevated C-reactive protein (Table 1). The AXR demonstrated stool loading in the right colon (Figure 1). After pain relief with analgesia, she was discharged with lactulose syrup.

**Table 1:** Results of the laboratory investigations

Parameter	Fourth day of pain	Eighth day of pain	Twelfth day of pain (admission day)	Second day after surgery	Fifth day after surgery	Reference value
C-reactive protein	3.85 mg/dL	2.42 mg/dL	0.77 mg/dL	19.51 mg/dL	3.17 mg/dL	0.0-0.5 mg/dL
Hemoglobin	10.6 g/dL	10.9 g/dL	10.8 g/dL	10.1 g/dL	9.8 g/dL	10.6-13.5 g/dL
White cell count	8.86 × 10^9^/L	9.08 × 10^9^/L	6.33 × 10^9^/L	10.8 × 10^9^/L	7.78 × 10^9^/L	4.37-9.68 × 10^9^/L
Platelets	262 × 10^9^/L	273 × 10^9^/L	300 × 10^9^/L	253 × 10^9^/L	359 × 10^9^/L	186-353 × 10^9^/L
Urea	21.4 mg/dL	19.26 mg/dL	-	14.98 mg/dL	6.42 mg/dL	15-40 mg/dL
Creatinine	0.74 mg/dL	0.70 mg/dL	-	0.63 mg/dL	0.58 mg/dL	0.57-1.11 mg/dL
Serum sodium	136 mmol/L	-	-	138 mmol/L	140 mmol/L	136-145 mmol/L
Serum potassium	4.1 mmol/L	-	-	3.4 mmol/L	3.4 mmol/L	3.5-5.1 mmol/L
Total bilirubin	-	0.4 mg/dL	-	0.5 mg/dL	0.4 mg/dL	0.2-1.2 mg/dL
Albumin	-	4.3 g/dL	-	2.7 g/dL	3.1 g/dL	3.5-5.2 g/dL
Alkaline phosphatase	-	67 U/L	-	45 U/L	76 U/L	40-150 U/L
Aspartate aminotransferase	-	21 U/L	-	18 U/L	19 U/L	5.0-34 U/L
Alanine aminotransferase	-	13 U/L	-	7 U/L	9 U/L	0.0-55 U/L
Gamma-glutamyl transferase	-	24 U/L	-	15 U/L	22 U/L	9.0-36 U/L
Glucose	-	90 mg/dL	-	-	-	60-139 mg/dL
Lipase	44 U/L	32 U/L	-	-	-	8.0-78 U/L

**Figure 2:**
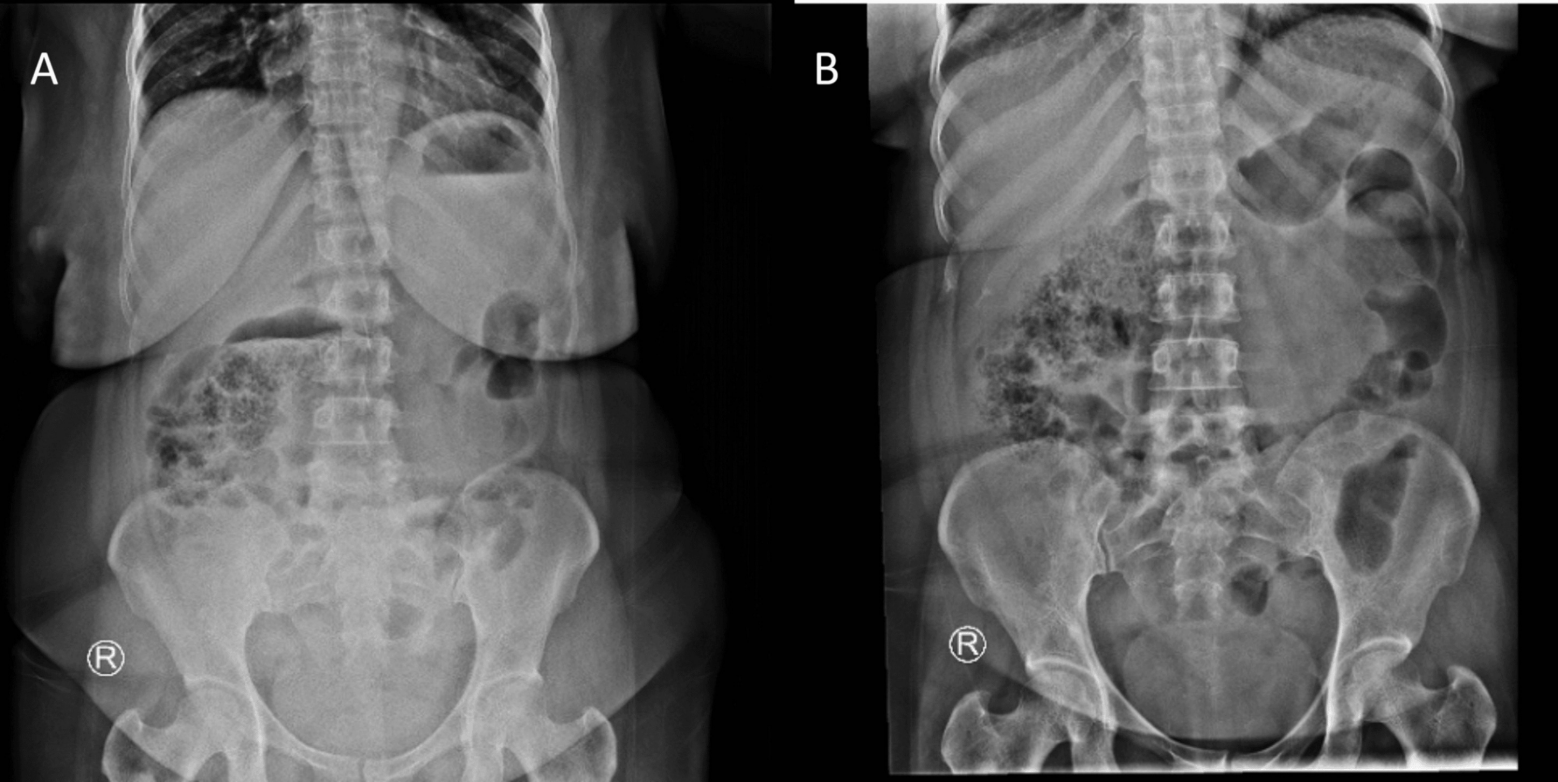
Second presentation abdominal X-ray (A) Erect; (B) Supine

The patient returned three days later (day eight of initial pain) with recurrent abdominal pain and new-onset diarrhea (five episodes in 24 hours). Repeat laboratory tests and AXR findings were unchanged (Figure 2, Table 1). Following symptomatic improvement with analgesia, she was discharged with instructions for an outpatient abdominal ultrasound.

**Figure 3:**
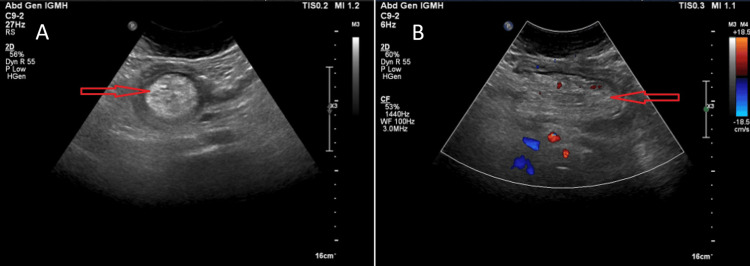
Abdominal ultrasound showing the intussusception (A) Target sign (red arrow); (B) Pseudokidney sign (red arrow)

**Figure 4:**
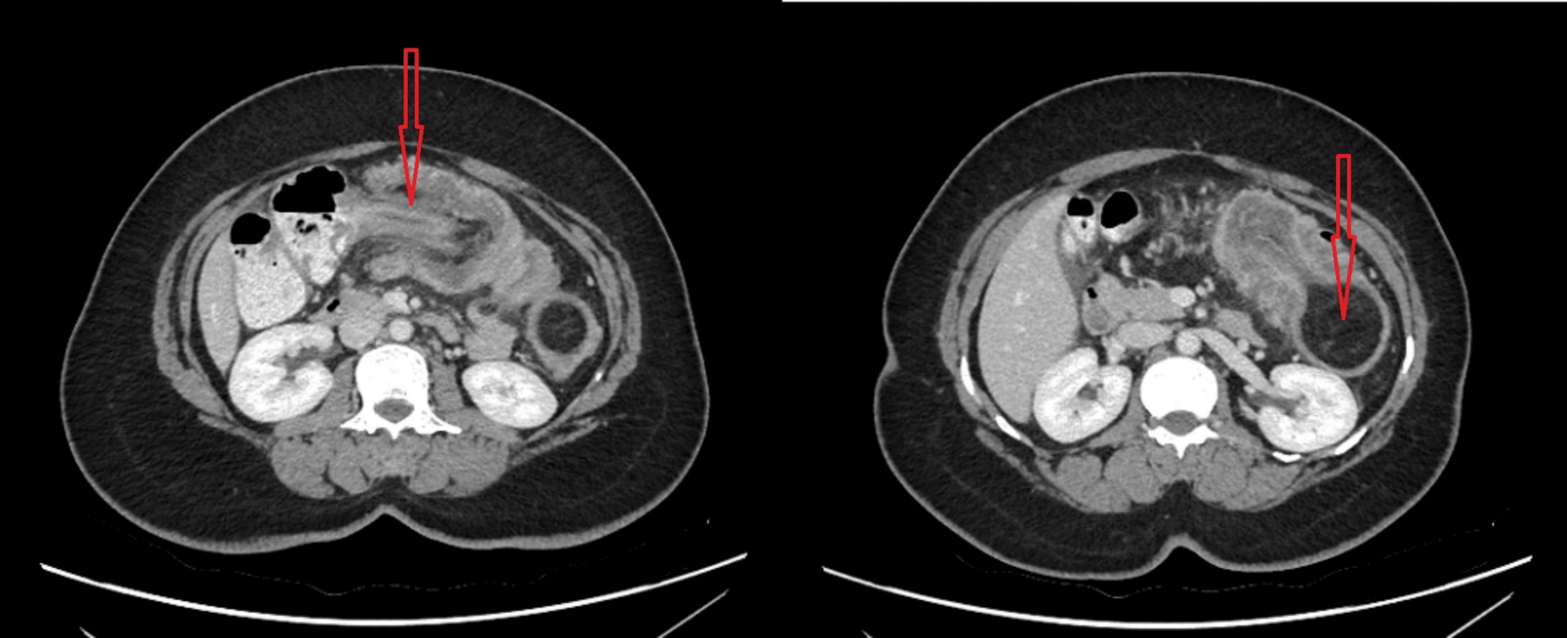
Abdominal CT scan showing colocolic intussusception (A) Intussusception of the transverse colon (red arrow); (B) Distal end of the intussusception showing the colonic lipoma (red arrow)

Four days later (day 12 of initial pain), ultrasound revealed an ileocolic intussusception, prompting hospital admission (Figure 3). The CT scan confirmed colocolic intussusception with a lead point exhibiting regular margins and fat density, most consistent with a submucosal lipoma. No enlarged lymph nodes were visible (Figure 4). Admission laboratory tests remained normal (Table 1). The only notable symptom at admission was abdominal pain exacerbated by defecation, with reported loose and frequent stools.

**Figure 5:**
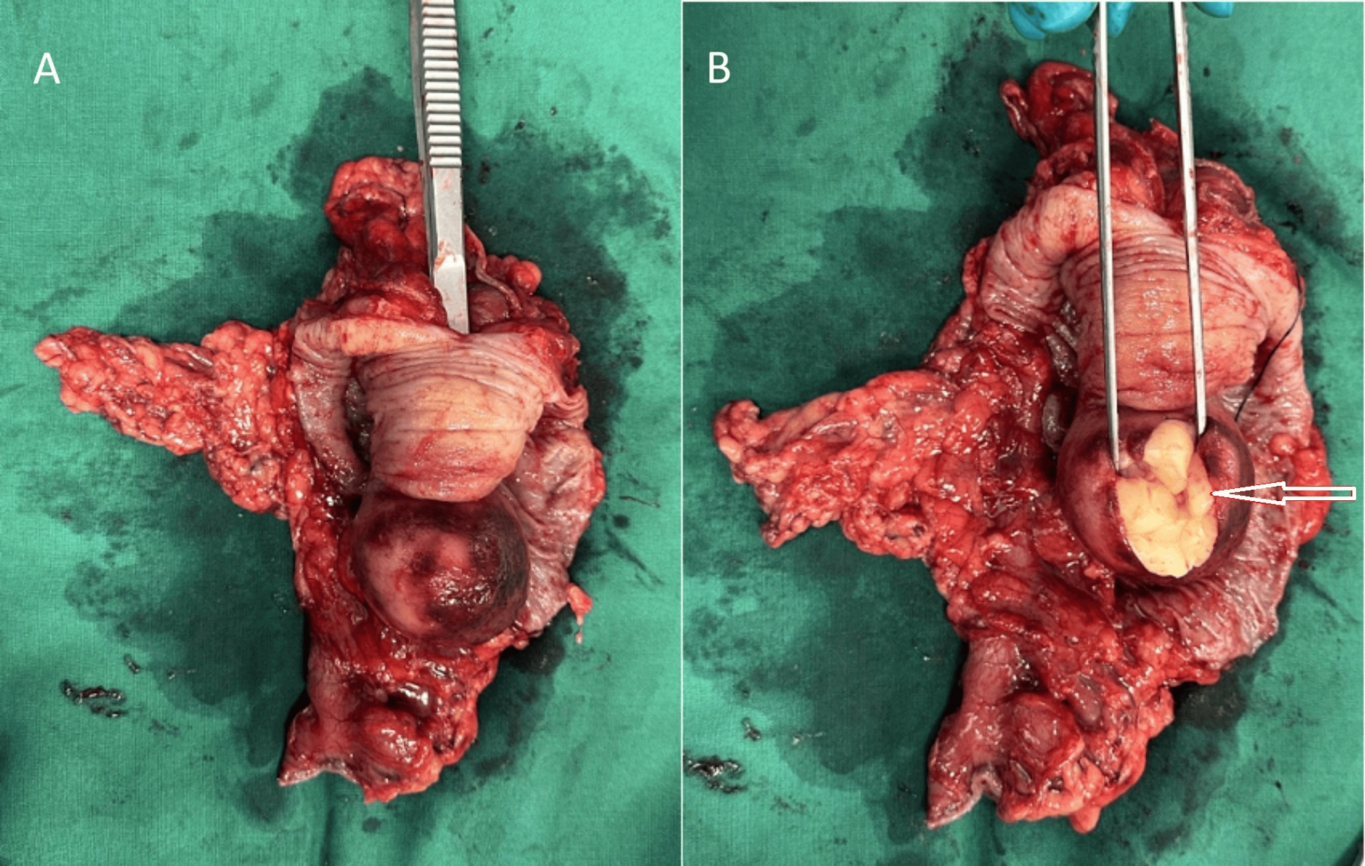
Surgical specimen (A) Intussusception incised; (B) Submucosal lipoma (white arrow)

The patient underwent midline laparotomy, which identified a colocolic intussusception involving the transverse colon (Figure 5). The lead point was a 5 × 5 cm soft, non-infiltrative submucosal fatty mass. No perforation, bowel gangrene, or palpable lymph nodes were noted. A 15 cm segmental resection was performed with side-to-side stapled anastomosis. The abdomen was closed in layers with placement of a 24F drain (Romsons, India) near the anastomosis.

Postoperative management included analgesia, intravenous fluids, and antibiotics for five days. A liquid diet was initiated on postoperative day two, and the surgical drain was removed on day five. Bowel function returned by postoperative day three; however, due to her chronic constipation, she did not have another bowel movement until postoperative day eight, when she was discharged. At her two-week follow-up, she reported no complaints, with a well-healed surgical wound, and her stitches were removed. Final histopathology confirmed a submucosal lipoma (5 × 4.5 × 4 cm) and an incidental serrated adenoma in the transverse colon. She was advised to undergo a colonoscopy for further evaluation of the incidental finding.

## Discussion

Intussusception in adults presents diagnostic challenges, as it typically manifests with nonspecific symptoms and, in rare cases, may even present as an extra-abdominal condition [3,8,11]. Abdominal pain, occurring in more than 90% of patients, initiates a broad differential diagnosis [3,7]. Without signs of intestinal obstruction, intussusception is often a low-priority consideration. We describe the case of a middle-aged, otherwise healthy woman with multiple ED visits for nonspecific intermittent abdominal pain. The presence of chronic constipation and stool shadowing on an AXR initially suggested fecal accumulation as the cause of her symptoms, further delaying the correct diagnosis. Studies report a mean symptom duration of 40 days for colocolic intussusception [3]. Our patient’s diagnosis after 12 days of symptoms falls within the subacute range reported in some series, highlighting the frequent diagnostic delay [1,3]. Cross-sectional imaging proves particularly valuable in such cases, as demonstrated in our report.

Colonic lipomas are a documented cause of adult intussusception. Pichioni et al. and Fiordaliso et al. reported 10 and 24 cases of colocolic intussusception secondary to colonic lipomas in their respective literature reviews [12,13]. The median ages were 48.5 years (range: 40-70) and 51.5 years (range: 29-82), respectively. The most common site for lipomas leading to intussusception was the ascending colon, accounting for 60% and 48% of cases, respectively, while transverse colon lipomas were found in 10% and 13% of cases, respectively [12,13]. Our case further supports the observation that colonic lipomas occur more frequently in middle-aged individuals; however, it occurred at a less frequent site. Lipomas account for 19-60% of reported colocolic intussusception cases [1,3,8,11]. The reported malignancy rate for the colocolic type is approximately 46% [14].

While CT scan remains the most sensitive diagnostic test for intussusception, ultrasound serves as a valuable screening tool [1,15]. Ultrasound offers several advantages: it is less time-consuming, involves no radiation exposure, and can be safely repeated. The presence of a target sign - a concentric pattern of hypoechoic and hyperechoic areas - or pseudokidney sign - a mass resembling a kidney with a central hyperechoic area surrounded by a hypoechoic region - on ultrasound is highly suggestive of intussusception (Figure 3). Ultrasound has an accuracy of approximately 49% [14]. However, its major limitations include bowel gas interference and reduced accuracy in obese patients [15]. CT scanning provides superior diagnostic capabilities, with an accuracy of 77.8%, as it can clearly demonstrate the lead point, characterize its nature, and precisely identify the involved bowel segment [1,2,14]. In our case, while ultrasound correctly identified intussusception, it failed to accurately localize the lesion. AXR may help detect bowel obstruction but lacks specificity for determining the underlying cause, with near-zero sensitivity for intussusception [8]. Barium enema can reveal characteristic cup-shaped or spiral defects, although this modality has been largely replaced by CT in modern practice [2].

Colonoscopy may be considered for stable patients to help differentiate between benign and malignant lesions, thereby assisting in surgical planning regarding the type of resection and the potential for lesion reduction [1]. In select cases, endoscopic treatment of the lead point may also be possible. However, this modality is contraindicated in patients presenting with acute obstruction [2,8]. In our case, colonoscopy could have provided additional diagnostic value by confirming the nature of the lead point and assessing the colon for other lesions. However, several factors supported proceeding directly to exploratory laparotomy: the high probability of a benign lesion based on CT findings, the absence of malignancy risk factors, limited evidence supporting colonoscopy's role in such cases, and the definitive need for surgical intervention given our lack of expertise in endoscopic resection of large submucosal lesions. In light of the incidentally detected serrated adenoma, we advised the patient to consider a screening colonoscopy for further evaluation of possible synchronous adenomas.

The choice between the laparoscopic and open approach is largely determined by the surgeon's experience, the availability of required instruments, and the patient's ability to tolerate pneumoperitoneum [2,8]. In this case, the laparoscopic approach would have been preferable, as it reduces postoperative pain, surgical site infection, and enhances recovery. However, due to the unavailability of certain instruments, such as laparoscopic staplers for intracorporeal anastomosis and a wound protector sleeve for extracorporeal anastomosis, along with the patient's overweight status and thick abdominal wall (which would have made extracorporeal anastomosis more challenging through a small incision), the open approach was chosen.

The other controversial issue is the attempt to reduce the intussusception. In general, if there is perforation, necrosis, or obvious malignancy, reduction is not recommended, and en bloc resection is the primary option due to the theoretical risks of contamination or intraluminal and/or intravenous tumor seeding [11]. The advantage of reduction is bowel length preservation, which is particularly important in cases of long intussusception involving the small bowel [2]. In the reported case, the affected segment was the transverse colon, approximately 15 cm in length. The remaining colon was sufficiently long to allow for a tension-free primary anastomosis. Despite preoperative imaging, patient history, and physical examination suggesting a benign lesion, the surgical team opted not to reduce the intussusception and proceeded with en bloc D1 resection, as bowel preservation was not deemed necessary.

The surgical team considered both segmental colectomy and extended right hemicolectomy. Given the low malignancy risk, supported by a negative family history, absence of constitutional symptoms, and CT findings consistent with a benign fatty tumor, segmental colectomy was selected as the optimal approach.

The patient’s postoperative hospital stay extended to eight days, exceeding the usual duration documented for uncomplicated cases [5]. While published series report average stays of 10-15 days, these predominantly involved sicker patients with complications [1,8,11]. In our case, the Enhanced Recovery After Surgery (ERAS) protocol was not followed strictly, and we acknowledge the need for improvement. Despite satisfactory recovery milestones - including the return of bowel function by postoperative day three and drain removal by day five - discharge could have been considered by day five, as her inflammatory markers had significantly decreased (Table 1). However, due to the patient’s reluctance to advance her diet, inadequate mobilization, and absence of bowel movements after day three, the surgical team opted for prolonged hospitalization.

## Conclusions

Intussusception is an uncommon cause of abdominal pain and may present with nonspecific symptoms, making its diagnosis challenging. Cross-sectional imaging plays an important role in diagnosis, particularly CT. Colonic lipoma is a common cause of colocolic intussusception, especially in middle-aged patients. Management varies from conservative treatment to en bloc surgical resection. In general, colocolic intussusception is usually managed with surgical resection due to the high incidence of lead points, which have a risk of being malignant.
